# An Insidious Gastrointestinal Bleeding from Secondary Aortoduodenal Fistula Leading to Septic Shock

**DOI:** 10.1155/2019/6261526

**Published:** 2019-05-13

**Authors:** Ahmad Khan, Ejaz Ahmad, Saad Javaid, Mohamed Riad Sankari

**Affiliations:** ^1^West Virginia University-Charleston Division, Charleston, WV, USA; ^2^Nishtar Hospital Multan, Pakistan; ^3^Charleston Area Medical Center, Charleston, West Virginia, USA

## Abstract

Insidious gastrointestinal bleeding from a secondary aortic fistula poses a significant diagnostic challenge. Failure to recognize it early on can lead to devastating outcomes. We describe a case of insidious gastrointestinal bleeding from a secondary aortic fistula in an elderly woman who presented with recurrent admissions for melanotic stools and eventually developed septic shock. Esophagogastroduodenoscopy did not reveal any obvious source of bleeding. The patient eventually had push endoscopy that revealed infected graft and a secondary aortoduodenal fistula. One should proceed with push enteroscopy in occult bleeding if the patient has a history of abdominal aortic aneurysm repair.

## 1. Introduction

A secondary aortoduodenal fistula (SAEF) is an uncommon complication of abdominal aortic aneurysms (AAA) repair surgeries [1]. Rarely, it manifests as insidious gastrointestinal bleeding that can lead to the infection of the graft; therefore, we suggest that a high index of suspicion as a failure to recognize it early on can result in considerable morbidity and mortality [2, 3]. We present a case of secondary aortoduodenal fistula after abdominal aortic aneurysm repair presented as slow recurrent gastrointestinal bleeding over several months leading eventually to infection of the graft and septic shock.

## 2. Case Report

**Figure 1:**
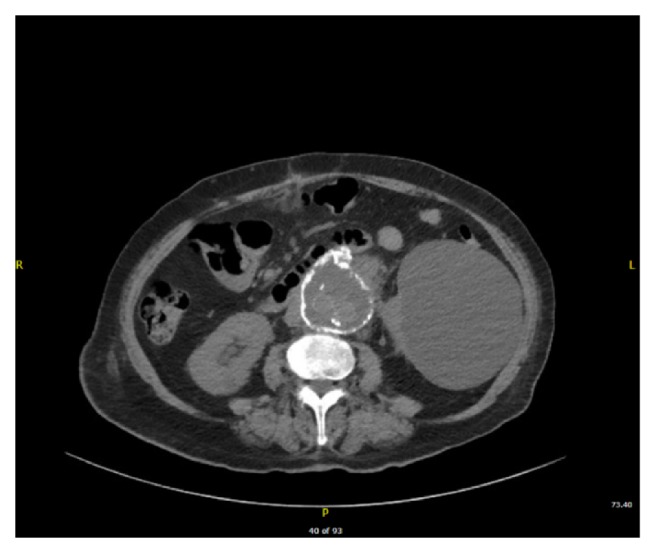
Computed tomography of abdomen showing nonspecific findings, irregularity of the “aneurysmal sac” with a small amount of fluid around the sac.

A 74-year-old female presented to the emergency department (ED) with upper abdominal pain and melanotic stools. She had an elective open juxtarenal abdominal aortic aneurysm repair a month before her index presentation. She was hemodynamically stable. Her pertinent initial labs showed a hemoglobin of 6.7 g/dl (baseline 9.6 g/dl) with a hematocrit of 23%. Patient did not have any fever or leukocytosis. A CT abdomen with contrast done in the ED for abdominal pain showed nonspecific findings, i.e, irregularity of the “aneurysmal sac” with a small amount of fluid around the sac (see Figure 1) which was read by the radiologist as early postsurgical changes. She was admitted and was started on proton pump inhibitors. An esophagogastroduodenoscopy (EGD) was performed that revealed mild duodenitis. Her hemoglobin remained stable the next couple of days, and she was discharged home with a 6-8-week course of proton pump inhibitors. Two months later, she presented again with similar complaints with a drop of hemoglobin. A repeat EGD was performed that did not reveal any obvious source of bleeding, and she was discharged home after stabilization.

**Figure 2:**
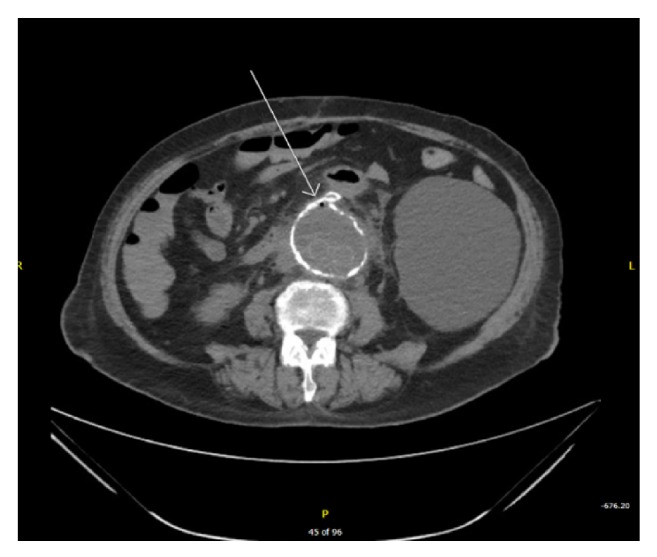
Computed tomography of abdomen showing tiny foci of air at the anterior aspect of the native aneurysm wrap just inferior to the location where the duodenum crosses.

**Figure 3:**
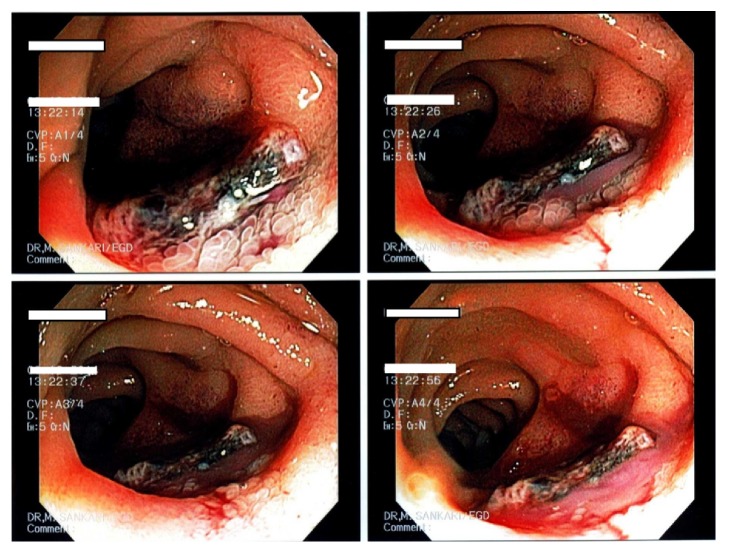
Push enteroscopy showing infected graft with purulent exudate extruding from an aortoduodenal fistula.

A month later, she came for the third time into the ED with abdominal pain, hematochezia, and profound hypotension. Her pertinent laboratory findings include leukocytosis, low hemoglobin and hematocrit, thrombocytopenia, and transaminitis. She was resuscitated with IV fluids and blood transfusions. She was started on broad spectrum antibiotics after blood cultures were drawn. A CT abdomen and pelvis was performed which showed tiny foci of air at the anterior aspect of the native aneurysm wrap just inferior to the location where duodenum crosses (see Figure 2). At that time, a decision was made to perform push enteroscopy instead of simple EGD to evaluate second and third portion of duodenum which showed an aortoduodenal fistula with infected graft adherent to the bowel wall and extruding purulent exudate (see Figure 3). She underwent emergent surgical excision of the infected graft and bypass grafting to restore vasculature. Her blood cultures and cultures from the graft revealed methicillin-resistant* Staphylococcus aureus* (MRSA) and* Streptococcus agalactiae*. Aggressive management was continued with proper antibiotics in the intensive care unit, but her condition deteriorated, and she expired within several days.

## 3. Discussion

Secondary aortoenteric fistula (SAEF) is an infrequent complication following any aortic reconstruction surgery. It results from mechanical erosion of the prosthetic graft or suture material into the surrounding bowel and sometimes even stent graft infection itself predisposes to erosion of the adherent bowel wall. The overall incidence reported is between 0.36% and 1.6% with the most common location to develop is the third and fourth parts of the duodenum due to its retroperitoneal location and proximity with the graft. The literature review demonstrated that more than 75% of cases are seen in the third and fourth part of the duodenum, followed by the 5% cases involving colon and less than 2% involving cecum. The risk factors include old age with median age of 65 years or above, male gender, and choice of procedure such as endovascular versus open and emergent versus nonemergent [3, 4].

The clinical presentation varies significantly and depends on the type of fistula, i.e., primary or secondary, and usually presents with gastrointestinal bleeding of any magnitude with melena in 54% and hematemesis in 41% cases, unexplained abdominal pain in 21% cases or graft infection and sepsis in 12% cases [5, 6]. The bleeding ranges from slow occult bleeding, i.e., minor herald bleed as seen in our patient, which can be self-limiting or can result into massive exsanguinating hemorrhage [7, 8]. The herald bleed poses a significant diagnostic challenge and can lead to graft infection and septic shock if not recognized early on as seen in our patient.

The diagnostic workup depends on the patient's hemodynamic status but should always be preceded with a detailed history and physical examination. A previous history of aortic reconstruction should always alert physicians about the possibility of SAEF associated bleeding. Computed tomography (CT) of the abdomen with contrast enhancement or a CT angiography (CTA) is the most frequent initial diagnostic test. The diagnostic yield of CT abdomen itself is low but can show any indirect evidence such as focal bowel wall thickening, gas or a collection surrounding the graft in case of infection [9–11]. CT mesenteric angiography (CTA) is superior to CT abdomen as it helps to visualize the aortic lumen itself and can demonstrate any extravascular collections or active contrast extravasation [12]. EGD is routinely done as a next step to rule out other sources of GI bleed. However, EGD can only localize the graft material or even pus inside the bowel only in less than 25% cases. However, its accuracy beyond the 2nd portion of the duodenum is limited, and a duodenoscope or push enteroscopy is used to evaluate the third and fourth parts of the duodenum [13–15]. In case of slow chronic bleed, the suture line or graft material can become a nidus of infection and can lead to sepsis and septic shock as seen in our case. When infection of the graft is suspected, 18-fluorodeoxyglucose positron emission tomography (18- FDG PET) scan or tagged white blood cell scan (WBC scan) can help to locate the source of infection. WBC scans have excellent sensitivity in the diagnosis of infected grafts in the early postoperative period, as 18- FDG PET scan can demonstrate false positive results [16–18].

The management of SAEF associated GI bleeding typically involves initial resuscitation, hemodynamic support, use of broad-spectrum antibiotics in case of infection, and early aortic repair [19]. An aortic repair is done through either open or endovascular approaches and includes vascular control, removal of infected or necrotic tissue, restoration of gastrointestinal continuity, and revascularization to restore the blood flow of the lower extremities. Endovascular options include endovascular balloon occlusion of the aorta, endovascular coil embolization, and stent-graft repair [20]. The major complications after an open repair include recurrence of fistula, aortic stump disruption, and recurrent infection. The prognosis is dependent on the early diagnosis and prompt surgical treatment which may prevent mortality of up to 80% [21].

The number of aortic reconstruction surgeries has been increasing due to improved screening methods and early diagnosis. Our case highlights the importance of maintaining a high index of suspicion even if the initial EGD is negative and one should proceed with push enteroscopy as failure to recognize it will lead to the devastating outcomes.
